# Fidelity-consistency and deliberateness of modifications in parenting programs

**DOI:** 10.1186/s43058-024-00545-4

**Published:** 2024-02-13

**Authors:** Kristoffer Pettersson, Pernilla Liedgren, Aaron R. Lyon, Henna Hasson, Ulrica von Thiele Schwarz

**Affiliations:** 1 School of Health, Care and Social Welfare, Mälardalen University, Västerås, Sweden; 2 Department of Psychiatry and Behavioral Sciences, University of Washington, Seattle, WA USA; 3 Procome Research Group, Department of Learning, Informatics, Management and Ethics, Medical Management Centre, Karolinska Institutet, 171 77 Stockholm, SE Sweden; 4 Unit for Implementation and Evaluation, Center for Epidemiology and Community Medicine (CES), Stockholm County Council, 171 29 Stockholm, SE Sweden

**Keywords:** Core components, Core functions, Modification, Adaptation, Fidelity, Parenting programs, Implementation

## Abstract

**Background:**

Evidence-based interventions (EBIs) are frequently modified in practice. It is recommended that decisions to modify EBIs should be made deliberately to ensure fidelity-consistency, yet the relationship between fidelity-consistency and deliberateness is not well understood. This study aims to explore modifications in a sample of practitioners delivering evidence-based parenting programs (i.e., interventions to strengthen parent–child relationships, reduce harmful interactions, and improve child health and well-being). The study investigated three research questions: (1) What kind of modifications are made during the delivery of parenting programs? (2) To what degree are the identified modifications consistent with the core functions of each program? and (3) Is deliberateness associated with the fidelity-consistency of the identified modifications?

**Methods:**

In total, 28 group leaders of five widely disseminated parenting programs in Sweden participated in five focus groups, and two participants from each group also participated in individual interviews (*n* = 10). A content analysis approach was used where the identification of modifications was directed by the Framework for Reporting Adaptations and Modifications-Enhanced (FRAME) and then assessed for fidelity-consistency and four levels of deliberateness (*universal*, *situational*, *conditional*, and *unintentional*). Chi-square tests were performed to compare consistent and inconsistent modifications, and logistic regression was performed to explore whether deliberateness predicted consistency.

**Results:**

A total of 137 content modifications were identified, covering most of the content modification categories in FRAME. The most common were *tailoring/tweaking/refining*, *adding elements*, *shortening/condensing*, *lengthening/extending*, and *integrating another treatment*. Modifications were mostly fidelity-consistent but consistency varied greatly among categories. Furthermore, modifications made *unintentionally* or *situationally* were more likely to be fidelity-inconsistent.

**Conclusions:**

These results indicate that explicit consideration of modifications and their impact could be essential for sustaining the fidelity-consistent use of EBIs, even as such interventions are continuously modified.

Contributions to the literature

1. This study provides insight into the types of modifications being made to evidence-based parenting programs, enhancing our understanding of how these interventions are used in local service settings.
2. Our findings elucidate the relationship between the deliberateness of modifications and fidelity consistency in evidence-based parenting programs, emphasizing the importance of explicit reasoning processes.
3. Our research underscores the value of the FRAME model as a tool for categorizing modifications and provides recommendations for further refinement of the framework.
4. These insights could help practitioners maintain fidelity while making adaptations to meet unique client needs and contexts in parenting programs.

## Background

Evidence-based interventions (EBIs) that address psychosocial problems are frequently modified in local practice settings [1]. Some modifications steer away from the intervention and might decrease client outcomes [2, 3], a phenomenon sometimes labeled *drift* [4]. Other modifications are made deliberately to improve fit with the local context and are referred to as *adaptations* [5, 6]. The term *fidelity* typically refers to the delivery of EBIs as intended by treatment developers [7]. However, modifications can be *fidelity-consistent* if they are made to preserve essential features of the intervention and *fidelity-inconsistent* if they *do not preserve these features* [8]. For example, cultural adaptation of an EBI could include changes to an intervention’s surface structure, such as using culturally relevant examples and concepts while preserving the essential features of the intervention [9, 10]. Thus, separating what type of modifications preserve essential features of an intervention is a crucial task in successfully managing modifications [11, 12].

Ideally, essential features of an EBI are established based on empirical findings that demonstrate causal relationships between certain activities (e.g., exposure activities in treating anxiety disorders) and outcomes (e.g., decreased anxiety response) [13]. However, knowledge of causal relationships between components and outcomes is generally lacking in psychosocial interventions [14]. Therefore, essential features are typically inferred from theory or fidelity measures used during program evaluation [15]. Although essential intervention processes are traditionally referred to as *core components*, recent publications argue that *core functions* is a more appropriate term, and that information regarding essential features is best summarized and organized accordingly [12, 16]. According to Perez Jolles et al. [16], “core functions are the core purposes of the change process that the health intervention seeks to facilitate” (p. 1033). Specific intervention functions can take many *forms*, meaning that they can be modified to serve needs in the local context and yet target the same core function. Hawe argues [17] that function-based standardization provides a means to modify interventions without sacrificing fidelity (i.e., it is a way to select fidelity-consistent modifications). Some initial attempts have been made to analyze the core functions of EBIs [18], yet, to our knowledge, no studies have used the concept of core functions to assess the fidelity-consistency of modifications made in local practice settings.

Although several models for guiding modifications exist [19, 20], the specific mechanisms involved in selecting fidelity-consistent modifications are poorly understood. Some argue that the *timing* of modifications is crucial, suggesting that *proactive* modifications (i.e., changes made in response to anticipated challenges) are more likely to be fidelity-consistent than those that are *reactive* (i.e., changes made in response to unanticipated challenges) [5, 8]. It is also argued that modifications selected using a systematic procedure are more likely to be fidelity-consistent than *unsystematic* or *unplanned* ones [20]. These decisions may be made by a team involved in a joint, proactive decision-making process at the early stages of implementation, before the EBI is implemented, and by individual practitioners making decisions when they are using the EBI in regular practice, prompting individual practitioners to consider modifications in more or less proactive and systematic ways. In doing so, practitioners draw upon cognitive processes that can be predominantly *intuitive* or *deliberate* [21]. Although research findings suggest that intuitive decisions are more prone to errors than deliberate ones [22, 23], to our knowledge, no studies have explored whether deliberateness is related to the fidelity-consistency of modifications.

This study aims to explore modifications in a sample of practitioners delivering parenting programs. Parenting programs are preventive psychosocial interventions that help parents and caregivers develop and improve their parenting skills while promoting positive child development and reducing or preventing behavioral, emotional, or developmental issues in children [24]. Most parenting programs are based on learning theory [25], social learning theory [26, 27], attachment theory [28], or some combination of these. Most follow a similar structure, including lectures, group discussions, and role-playing exercises. Parenting programs are among the most thoroughly studied preventive psychosocial interventions, with several large-scale meta-analyses showing robust and sustained effects [29, 30]. These programs also make an ideal case for studying modifications, as they (1) are well disseminated in local community settings; (2) are highly structured, with accompanying protocols that group leaders are expected to adhere to; (3) have a well-established evidence base [31]; and (4) are frequently modified in practice, especially for cultural reasons [10]. The study investigated three research questions: (1) What kind of modifications are made during the provision of parenting programs? (2) To what degree are the identified modifications consistent with the core functions of each program? and (3) Is deliberateness associated with the fidelity-consistency of the identified modifications?

## Methods

### Study design

The study employed a hybrid of directed and summative content analysis [32]. Data were collected through focus groups and semi-structured individual interviews to ensure a rich palette of examples of modifications that group leaders of parenting programs had made in practice (research question 1). These identified modifications were subsequently coded for fidelity to core functions and deliberateness and analyzed quantitatively to explore research questions 2 and 3.

### Participants

The study included 28 group leaders from diverse professions, such as social work, preschool teaching, and psychology. They represented five of the most widely disseminated parenting programs in Sweden: All Children in Focus (*n* = 8), Comet (*n* = 7), Triple P (*n* = 6), Connect (*n* = 4), and COPE (*n* = 3). The average participant age was 50.5 years (*SD* = 11.4, range = 31–68). They had an average of 6.36 years of experience working with parenting programs (*SD* = 4.17, range = 1–18) and had led an average of 12.11 parenting groups (*SD* = 9.28, range = 2–35).

### Recruitment

The recruitment was carried out in four steps. First, stratified purposeful sampling [33] was used to select 30 of Sweden’s 290 municipalities. Public records were consulted to categorize municipalities into three strata based on size (small, medium, and large) and two additional strata based on geographical characteristics (urban and rural). This stratification was intentionally designed to ensure a comprehensive representation of the diversity in municipality characteristics relevant to the implementation of parenting programs. Second, municipal websites were examined to assess what types of parenting programs were offered. At this juncture, eight small municipalities were excluded from the sample, as they did not offer any of the five targeted parenting programs under investigation. Third, the remaining 22 municipalities were contacted, 17 responded, and an initial meeting was held to describe the study further. After this meeting, eight agencies, covering large and medium municipalities in rural and urban areas, agreed to participate in the study. Fourth, individual group leaders were contacted, and interviews were scheduled after they agreed to participate in the study.

Participants were divided into groups of four to eight people based foremost on the program in which they had trained (one group was mixed, while three participants were from COPE and two from Comet). Five focus groups were conducted and organized around questions intended to stimulate open-ended discussions about modifications that group leaders had encountered in their work. Sample questions are as follows: “What do you do when it is problematic to adhere to the program?” and “Have there been times when you changed something in the program? What did you do?”.

After the initial round of focus groups, 10 additional interviews (two group leaders from each focus group) were conducted to provide elaborations and more detailed descriptions of modifications. To cover the range of experiences in the groups, the group leaders with the least and most experience working with parenting programs were invited to participate. Our rationale was that focus-group dynamics can highlight experienced voices, possibly overshadowing those with less experience. Additionally, focus groups may carry a subtle social desirability bias, making it valuable to include a broader range of experience in follow-up interviews. The interviews were more structured than the focus groups and designed to obtain specific examples of content modifications included in FRAME [8]. Example questions include the follows: “Can you give more examples of changes, improvements, or adjustments to the program that you have made or thought about making?”, “Do you have additional examples of adding or skipping parts of the program?”, and “Have you ever changed the order of any of the interventions?”.

Interviews were conducted in Swedish by one of the authors (K. P.), with a second author (P. L.) participating in focus groups 1 and 2. The meetings were held on an online meeting platform (Zoom), and the video was recorded locally using third-party software (VideoSolo). Each focus group was 90 min long, and the average duration of individual interviews was 36 min (ranging from 25 to 48 min).

The study was approved by the Swedish Ethical Review Authority (Dnr 2021–00832). Participants received oral and written information about the purpose of the study, what participation entailed, that no identifying information would be reported, and that they could withdraw their consent at any time without further explanation. All participants gave their informed consent in writing before the interviews started.

### Analysis

Interviews were transcribed verbatim, and three authors (K. P., P. L., and U. v. T. S.) collaborated throughout the analysis phase. Consensus coding was applied, and disagreement was resolved through discussion [34]. The interviews were first analyzed using a directed content analysis approach in which the modification forms were classified [32] based on FRAME [8, 35]. The data extracted were then coded and quantitively analyzed using chi-square analyses to compare fidelity-consistent and -inconsistent content modifications. In addition, logistic regression was used to investigate the relationship between deliberateness and modification consistency.

#### Assessment of content modifications

The content modification section of FRAME [8, 35] was used to categorize the forms of modifications represented in the data. To undertake a separate analysis of form and function, we could not use all content modification categories precisely as specified in FRAME. Specifically, according to Stirman et al. [35], *adding elements* and *tailoring/tweaking/refining* should be used for modifications that keep intervention mechanisms unchanged (i.e., that are fidelity-consistent). Thus, these specific categories presuppose fidelity-consistency, although there might also be fidelity-inconsistent modifications that have the form of adding or tailoring (e.g., adding a component from another, theoretically incompatible, program). In FRAME, these modifications would likely be coded as *departing from the intervention with/without returning (drift)*, omitting the information about the form. As the aim of the study required form and function to be assessed separately, the content modification section of FRAME was used exclusively to categorize modification forms. This allowed the coding of *tailoring/tweaking/refining* and *adding elements* regardless of whether the changes were fidelity-consistent or not. The category labeled *departing from the intervention with/without returning (drift)* was not used since it does not specify a modification form.

#### Assessment of fidelity-consistency

To assess whether identified modifications were fidelity-consistent or -inconsistent, we followed a three-step procedure outlined by Kirk et al. [36]. First, EBI materials for all parenting programs were reviewed by one author (K. P.), including manuals/protocols and key publications describing the program’s theory of change or logic model. To identify core functions, we relied on the definition provided by Pérez et al. [37]: “core functions are the core purposes of the change process that the health intervention seeks to facilitate” (p. 1033). In most cases, the published materials included descriptions of core purposes corresponding to the definition of Pérez et al. When there were gaps or inconsistencies in the written material, we reached out to local program managers or researchers with expert knowledge of the specific program to decide which description best reflected the core functions of the programs. Second, the core functions and forms were listed for each program (Table 1). The intervention forms were also extracted and exemplified from the various training activities included in the training manuals. In the third and final step, three authors (K. P., P. L., U. v. T. S.) collaborated on assessing the fidelity-consistency of each content modification by considering whether the modification in question could achieve any of the core functions specified for the program. If so, the modifications were coded as fidelity-consistent; if not, they were coded as fidelity-inconsistent.

**Table 1:** Characteristics of parenting programs, theoretical underpinnings, core functions, and example forms. Functions for each program were extracted from: All Children in Focus [38, 39] Comet [40], Connect [41], Cope [42], and Triple P [43]

Programs	*Target age group*	*No. of sessions*	*Session duration (hours)*	*Theory*	*Goals or aims of the programs (i.e., functions)*	*Example of activities (i.e., forms)*
All Children in Focus	3–12	4	2.5	Behavior analysis Social learning theory	• Increase positive attention and warmth • Provide positive feedback and reinforcement of positive behaviors • Increase understanding of what maintains child behavior • Reduce harsh parenting • Increase consistent parenting • Reduce unnecessary conflicts • Increase conflict management skills	Didactive teaching (e.g., parents as role models, child-directed play) Video illustrations (e.g., how to give positive attention, stressful morning routines) Group work (e.g., practice analyzing behaviors, how to deal with challenging situations)
Comet	3–12	11	2.5	Behavior analysis Social learning theory	• Increase positive attention and warmth • Strengthen parents’ ability to recognize and encourage positive behaviors • Set limits and consistent parenting • Reduce unnecessary conflicts • Teach collaborative problem-solving • Increase appropriate supervision	Didactive teaching (e.g., attention as reinforcement, how to validate emotions) Video illustrations (e.g., how to give positive feedback, children imitate their parents) Role-plays (e.g., task shifting, deescalate conflicts)
Connect	8–18	9	1.5	Attachment theory Systemic theory Relational theories	• Reduce parents’ feelings of distress • Develop reflective capacity and awareness of attachment • Increase parents’ capacity to provide a safe haven and secure base • Strengthen the parent-teen bond and protect from adversity	Didactive teaching (e.g., what is attachment, balance attachment, and autonomy) Illustrative role-plays by group leaders (e.g., responsive and nonresponsive ways of interacting) Reflection exercises (e.g., needs through the life span, what gets us stuck in negative relationship patterns)
Cope	3–12 and 12–18	7 and 10	2	Social learning theory Social-cognitive psychology Family systems theory Theories of Group Processes	• Increase supportive communication • Encourage positive behavior • Improve family relationships • Avoid conflicts • Manage transitions • Negotiate and solve problems	Didactive teaching (e.g., how to improve connection, distinguish small and big problems) Video illustrations (e.g., common parenting mistakes, collaborative planning) Group discussions (e.g., recognize problematic interaction patterns, how to stay calm as a parent)
Triple P	0–5	4^a^	2	Child and family behavior therapy Behavior analysis	• Ensure a safe and engaging environment • Create a positive learning environment • Teach assertive discipline • Encourage realistic expectations • Promote parents’ self-care	Didactive teaching (e.g., causes of behavior, how to encourage positive behavior) Group discussions (e.g., defining positive parenting, how to be a good role model) Role plays (e.g., task switching, support skill development)

^a^Triple P uses a graded format in five levels (light universal to more intense indicated interventions). In Sweden, Triple P is available in three levels: seminar series, individual counseling, and groups. The group level consists of four group sessions, followed by four individual sessions. Only the group sessions are targeted in this study

The research team encompassed specialized expertise pivotal for the assessments made, including two licensed clinical psychologists with extensive knowledge of the theoretical underpinnings of the programs (K. P. and U. v. T. S.), a licensed clinical child psychologist with significant experience with parenting programs (A. L.), and a Child and Family social worker with extensive practical experience in the field (P. L.). Together, this ensured a robust and informed analysis of program modifications against core functions.

#### Assessment of deliberateness

To assess the deliberateness involved in modifications, we constructed a coding scheme based on Bennet-Levy’s model of practitioner skill acquisition and refinement [44]. According to this model, practitioners refine their procedural rules by noticing and reflecting on the mismatches between current knowledge and the challenges presented in clinical situations. Applied to modifications, we conceptualized four levels of deliberateness ranging from carefully considered to unintended: (1) *universal*: carefully considered modifications that extend over all sessions, (2) *conditional*: prospectively articulated strategies for modifications in response to circumstances that sometimes arise, (3) *situational*: modifications made to resolve any spontaneously arising difficulty, and (4) *unintentional*: unintended, involuntary, or accidental modifications made without any apparent reason in mind. Each identified content modification was assessed through collaboration between three authors (K. P., P. L., U. v. T. S.).

## Results

### Research question 1: content modifications

**Fig. 1:**
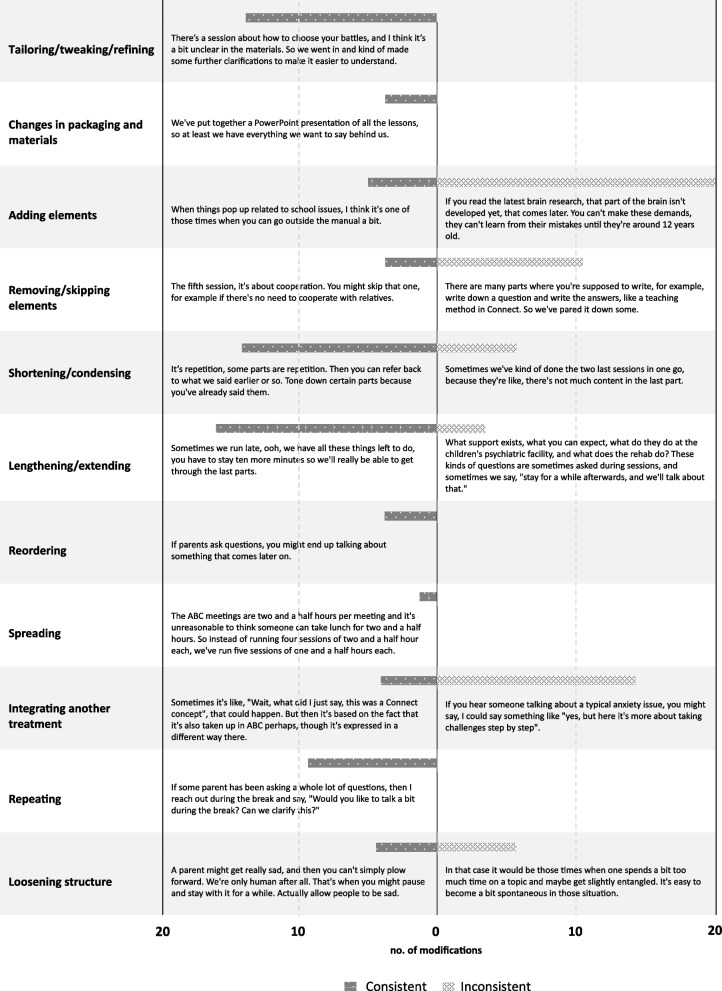
Fidelity-consistent and inconsistent content modifications, including sample quotes. The bars represent the number of modification examples identified in each category from 0 to 20 (no category had more than 20 examples). The sample did not include examples of *substituting* or *integrating into another framework*. *Departing from the intervention with and without returning* was not used in the categorization since they include fidelity-consistency specification in their definition

We identified 137 examples of fidelity-consistent and -inconsistent modifications in 11 of the 13 categories in FRAME (Fig. 1). The most common examples of modifications were *tailoring/tweaking/refining*, *adding elements*, *shortening/condensing*, *lengthening/extending*, and *integrating another treatment*.

#### Tailoring/tweaking/refining

The group leaders described several minor adjustments made to address the specific needs of parents and their children. Modifications in this category were typically clarifications, elaborations, and changes in the way aspects of the content were emphasized.

During the pandemic, when groups were provided online, group leaders also tweaked some presentation elements, for example, relying more on lectures than role-playing or discussions. These changes were also coded as consistent since they were made to facilitate delivery in this new format.

#### Changes in packaging and materials

This category only included three fidelity-consistent modifications that were made to make teaching materials easier to understand. First, in one of the programs, the group leaders made a PowerPoint presentation to support delivery. The second example was a modification of a point system that was made more age appropriate than the original version. Third, one group leader made an illustration that summarized the program’s conception of “good parenting.”

#### Adding elements

Additions were typically made following a direct request for tips, ideas, and advice, or they included information that the group leaders viewed as essential additions. Fidelity-consistent modifications were, for example, guiding parents in monitoring their children online or providing support in communicating with the school. Fidelity-inconsistent modifications included advising on health-related topics not implied by the programs core functions, suggesting reading materials on topics unrelated to the program, and sharing research findings that sometimes conflicted with the program content.

Group leaders working with immigrant families also provided information about Swedish society or how to manage governmental contacts. However, since this information is not included in the original program and is unrelated to the parental role, these examples were coded as inconsistent.

#### Removing/skipping elements

Group leaders gave examples of skipping specific exercises, role-plays, or group discussions. Many of these were assessed as fidelity-inconsistent, but sometimes there was a clear rationale for why the specific intervention was not needed. For example, a group leader justified the removal based on the evident lack of need for a specific element in one of the groups (Fig. 1). However, most examples in this category involved removing necessary elements, such as excluding token-economy instructions or didactic role plays and were thus coded as inconsistent.

#### Shortening/condensing

Several slight reductions in the time spent on specific exercises were reported, for example, spending less time on repeating sections of the material if everyone seemed to be following (Fig. 1). When examples of shortening/condensing were more significant, they were coded as fidelity-inconsistent, such as merging two sessions to save time or spending little time on elements at the end of the session.

#### Lengthening/extending

Group leaders either extended the session time or some part of the session or gave some parents the opportunity to stay afterward. These modifications were coded as consistent if the extension included clarifications or elaborations on already presented themes. In contrast, examples were coded as fidelity-inconsistent when the matters covered strayed from the material of the program (Fig. 1) to topics unrelated to the core functions of the programs.

#### Reordering

Three examples of reordering were identified. These were coded as fidelity-consistent and included examples of introducing certain concepts or ideas earlier, for example, guiding participants on responding to children’s emotions, even though that specific component was yet to come.

#### Spreading

The material contained one example of spreading: giving the program over five sessions instead of four to allow parents to participate during lunchtime. This modification was coded as consistent since no content was changed.

#### Integrating another treatment

Group leaders integrated treatments by arranging individual parallel contacts, integrating concepts or strategies from other parenting programs, or integrating treatment content unrelated to parenting. This modification was sometimes targeted to individual parents’ needs but could also result from group leaders’ knowledge and interest. If the content of other treatments was consistent with the core functions of the evaluated parenting programs, such as when group leaders used concepts or activities from other parenting programs with similar theoretical assumptions and goals, modifications were coded as fidelity-consistent; otherwise, they were coded as fidelity-inconsistent.

#### Repeating

All repetitions were fidelity-consistent and typically consisted of individual meetings during breaks and before, after, or between scheduled group sessions. The group leaders conducted these meetings based on the needs of the individual parents or because someone had not attended a session and needed some support to catch up with the others. Some of these examples were also coded as lengthening/extending.

#### Loosening structure

Some group leaders described their preferred style as somewhat looser than that prescribed in the manuals. This could result in inconsistent modifications if they became caught up in a discussion and failed to provide other content in line with core functions (Fig. 1). Other described situations that required them to depart from the structure included it being deemed inappropriate to interrupt valuable processes linked to core functions of the programs. Most of the group leaders said that it is essential to strike a balance between flexibility and rigidity.

### Research question 2: fidelity-consistency of modifications

Comparison between fidelity-consistent (*n* = 78, 57%) and fidelity-inconsistent (*n* = 59, 43%), modifications showed that fidelity-consistent modifications were significantly more common than fidelity-inconsistent modifications to be reported in focus groups and interviews, *χ*^2^ (1, *N* = 137) = 2.64, *p* = 0.011.

Separate chi-square tests compared consistent and inconsistent modifications for those modification categories that demonstrated both types (Table 2). *Lengthening/extending* included significantly more fidelity-consistent modifications, while *adding elements*, *removing/skipping*, and *integrating another treatment* included significantly more fidelity-inconsistent modifications. In *shortening/condensing* and *loosening structures*, no significant differences were found.

**Table 2:** Chi-square tests comparing fidelity-consistent and inconsistent content modifications

	*Consistent*	*Inconsistent*	*Chi-square*	*p*-*value*
*Adding elements*	5	20	*χ*^2^ (1, *N* = 25) = 9	*p* = .003
*Removing/skipping*	3	11	*χ*^2^ (1, *N* = 14) = 4.571	*p* = .033
*Shortening/condensing*	14	6	*χ*^2^ (1, *N* = 20) = 3.2	*p* = .074
*Lenghthening/extending*	17	3	*χ*^2^ (1, *N* = 20) = 9.8	*p* = .002
*Integrating another treatment*	4	13	*χ*^2^ (1, *N* = 17) = 4.765	*p* = .029
*Loosening structure*	5	6	*χ*^2^ (1, *N* = 11) = .091	*p* = 0.763

### Research question 3: relationship between deliberateness and fidelity-consistency

A logistic regression analysis was performed with fidelity-consistency as the dependent variable and the four levels of deliberateness as a categorical predictor variable. The 137 examples of modifications were analyzed. The model explained 16% of the variance in the consistency scores and the Omnibus *χ*^2^ (3) value of 17.37 (*p* < 0.001), suggesting that the model as a whole was a good fit for the data (Table 3).

**Table 3:** Results of logistic regression using consistency as the dependent variable and the four levels of deliberateness as predictor variables

				*95% CI*		
	B (SE)	Wald	Sig.	Lower	Odds ratio	Upper
*Universal*		12.92	.005	1	1	1
*Conditional*	1.14 (0.61)	3.46	.063	0.94	3.12	10.36
*Situational*	1.51 (0.63)	5.78	.016	1.32	4.54	15.56
*Unintentional*	3.26 (0.95)	11.92	< .001	4.01	26.13	166.6
*Constant*	− 1.56 (0.55)	8.02	.005			

*Note: B* = unstandardized regression coefficient; *SE* = standard error of the coefficient; Wald = Wald, chi-square statistic; Sig. = significance level (*p*-value); 95% CI = 95% confidence interval for the odds ratio; Lower = lower bound of the 95% CI; Odds ratio = odds ratio of the predictor variable; Upper = upper bound of the 95% CI; *R*^2^ = 0.16 (Nagelkerke). Omnibus *χ*^2^ (3) = 17.37, *p* < .001

Since the presence of fidelity-consistent modifications was assumed to be higher among modifications that had been carefully considered, *universal* was used as the reference category. Modifications made *conditionally* (clearly articulated strategies in response to circumstances that sometimes arise) were not significantly more likely to be fidelity-inconsistent. However, modifications made *situationally* (modifications made to resolve spontaneously arising difficulties) and *unintentionally* (involuntary or accidental modifications made without any apparent reason) showed an increased odds of being fidelity-inconsistent. The most substantial effect was found in modifications coded as unintentional, which increased the odds of fidelity-inconsistent modifications 26.13 times.

## Discussion

We identified 137 examples of content modifications across FRAME categories, the most common being *tailoring/tweaking/refining*, *adding elements*, *shortening/condensing*, *lengthening/extending*, and *integrating another treatment*. The proportions of consistent and inconsistent modifications varied greatly among categories, with six categories having only, or significantly more, fidelity-consistent modifications and three having significantly more fidelity-inconsistent modifications. Modifications made without apparent reason (i.e., *unintentionally*) or to resolve spontaneous arising difficulties (i.e., *situationally*) were more likely to be fidelity-inconsistent than those that were carefully considered.

Although we did not find examples of modifications in all categories of FRAME [8, 35], we found that the framework provided a helpful structure that was well-suited to identify content modifications in the context of parenting programs. For methodological reasons, we included both fidelity-consistent and -inconsistent examples of the categories *adding elements* and *tailoring/tweaking/refining*, even though the original definitions of these refer only to fidelity-inconsistent modifications [35]. Thus, we treated these two categories in the same way as the other FRAME categories, enabling separate assessments of modification forms and functions. Using this approach, we found that most examples of *adding elements* were fidelity-inconsistent. Had the assessment been made as defined in FRAME, these modifications would have been classified as *departing from the intervention* and mixed with other examples. Although this might be sufficient for some research applications, information about the form of the modification would have been lost. Similarly, it has been proposed that *removing elements* should generally be considered fidelity-inconsistent [45], but the findings from the current study paint a more nuanced picture and show both consistent and inconsistent ways of removing elements. Although there may be theoretical reasons to expect certain modifications to be primarily fidelity-consistent or -inconsistent, whether that is indeed the case is an empirical question, and the findings from this study indicate that *adding elements* and *removing elements* contain both fidelity-consistent and -inconsistent examples. Thus, our findings imply that a future iteration of FRAME might benefit from separating functions in terms of fidelity-consistency/-inconsistency from forms (i.e., from the categories). FRAME currently allows for tracking the fidelity consistency of modifications, but further separation from the nature of the modifications would enable more neutral- and context-sensitive assessments. Moreover, service providers could benefit from this distinction, as they often enact additional functions not explicitly included in specific programs, such as providing educational support in their role as teachers. Such a modification may be deemed fidelity-inconsistent within the narrow scope of one program but align with broader service functions. Currently, FRAME’s flexibility is limited in assessing these intricate interplays. While this will not impede every implementation project using FRAME, further development in these areas would enhance its utility, especially in applied settings.

Among the identified modifications, we found that less deliberate alterations were more likely to diverge from program fidelity than their more deliberate counterparts. This finding is in line with Moore et al. [5], who showed that proactive modifications made in naturalistic settings were more likely to align with the program’s goals and theories than reactive modifications. These findings suggest that the reasoning processes involved in making modifications could play a key role in determining the eventual success of interventions throughout their life cycle. Although our data did not specifically explore organizational practices encouraging such deliberateness, anecdotal observations indicated varying degrees of openness to discussing the topic in different work settings. Implementation strategies designed to increase the adoption and large-scale use of EBIs by promoting adaptability, which involves identifying the ways a clinical innovation can be tailored to meet local needs [46], will likely be most effective when they can maximize the deliberateness of those processes. This might be achieved by carving out time to discuss an intervention’s core functions or building in reflective opportunities during which implementers can explicitly consider the alignment of an intervention with the needs and constraints of their contexts.

### Limitations

We relied on interview data to obtain examples of modifications, which had both pros and cons. We found that group leaders sometimes needed extended and repeated questioning to express certain modifications they made. Additionally, there was some confusion among the group leaders about what constituted a modification. Despite these challenges, the interview format allowed us to capture many modifications that might otherwise have gone unreported. However, there were also drawbacks to this approach. Some group leaders expressed concern that modifications were a sensitive topic, implying that social desirability could have made group leaders withhold examples of fidelity-inconsistent modifications [47]. This concern could explain why we identified more fidelity-consistent modifications, while Moore et al. [5], who used surveys instead of interviews, identified a more significant proportion of fidelity-inconsistent modifications. However, it is also possible that our findings are skewed toward consistent examples because group leaders failed to report fidelity-inconsistent modifications due to more of this type being unintentional and thus harder to remember [48]. Future studies could combine direct observation with methods inspired by natural decision-making [49].

To assess content modifications, we relied on the well-established coding scheme FRAME [8, 35] in combination with a procedure for assessing fidelity-consistency to core functions [36] and our own approach to assessing deliberateness. Our data did not possess the granularity required to discern meaningful program-level differences in modifications. Further exploration of the nuances of modifications in parenting programs is much needed. We also note that there is a great need for further methodological development in this area. Reliable procedures for the identification of core elements are a prerequisite for producing a coherent science of modification options and outcomes.

#### Implications

Supporting fidelity-consistent modifications remains a goal throughout an EBI’s life cycle. To increase the likelihood that iterative modifications will be in line with interventions, service providers could act to directly support deliberate reasoning about reasons for modification, modification options, and their potential influence on outcomes. For example, in the context of parenting programs, service providers could create opportunities for group leaders to reflect on their actions and decision-making processes through regular supervision, feedback sessions, or training in understanding the theoretical underpinnings and core elements of the intervention [50]. Over time, such opportunities could enhance the group leaders’ understanding of the intervention and how to manage modification concerns, such as whether they should aim for fidelity-consistent modification or deliberately choose fidelity-inconsistent options if needed.

FRAME has helped to usher in an era in implementation science where modifications are routinely surfaced, acknowledged, and explored. Researchers relying on FRAME [8, 35] to classify content modifications should be aware that issues regarding fidelity-consistency may be implied in several categories, especially if they are investigating both intervention forms and functions. In such instances, and based on the findings of this study, we suggest that the content modification section of FRAME might be used exclusively to classify the modification forms, and that separate procedures could be employed to assess the essential elements of an EBI.

## Conclusions

This study adds to the current conceptualization of modifications and fidelity as coexisting phenomena. By assessing the fidelity-consistency of modifications in parenting programs and the degree of deliberateness involved, we found that deliberate modifications were more likely to be fidelity-consistent. Although based on a small sample, the results indicate that deliberate reasoning on modifications could be vital for the sustainment of fidelity-consistent evidence-based practices.

## Data Availability

The datasets used will be available from the corresponding author upon reasonable request.
